# Preserved autoregulation of coronary flow after off-pump coronary artery bypass grafting: retrospective assessment of intraoperative transit time flowmetry with and without intra-aortic balloon counterpulsation

**DOI:** 10.1186/s13019-016-0550-8

**Published:** 2016-11-28

**Authors:** Hiroyuki Nakajima, Atsushi Iguchi, Mimiko Tabata, Masaru Kambe, Masahiro Ikeda, Kazuhiko Uwabe, Toshihisa Asakura, Hiroshi Niinami

**Affiliations:** Department of Cardiovascular Surgery, Saitama Medical University, International Medical Center, 1397-1 Yamane, Hidaka, Saitama 350-1298 Japan

**Keywords:** Off-pump, CABG, Surgery, Autoregulation, Graft flow

## Abstract

**Background:**

Intra-aortic balloon pumping (IABP) markedly increases graft flow after coronary artery bypass grafting (CABG) with cardiopulmonary bypass. We sought to delineate the effects of IABP on graft flow after off-pump CABG (OPCAB).

**Methods:**

The clinical records of 32 patients (25 male, 7 female; mean age: 70 ± 9 years) who underwent OPCAB with IABP between January 2011 and May 2015 were retrospectively reviewed. Thirteen patients (41%) had a history of myocardial infarction, and 13 patients (41%) had a history of percutaneous coronary intervention. In total, there were 76 bypass grafts with 102 distal anastomoses. These included 50 in situ or pedicled grafts and 26 aortocoronary grafts. After completion of the anastomoses, the heart was positioned normally, and graft flow with IABP was measured using transit-time flowmetry under stable circulation. Then, IABP was turned off for 30 s to a few minutes, until graft flow was constant, for measurement of flow off IABP.

**Results:**

The angiographic patency rate was 100% (47/47). Overall, graft flow was 55 ± 36 ml/min on IABP and 53 ± 36 ml/min off IABP (*p* = 0.37). The pulsatility index was 4.1 ± 2.1 on IABP and 2.7 ± 1.5 off IABP (*p* < 0.001). There was no significant difference in graft flow between on and off IABP for aortocoronary bypass or in situ grafts. Graft flow was 57 ± 36 ml/min on IABP and 55 ± 37 ml/min off IABP (*p* = 0.41) in in situ grafts and 52 ± 34 ml/min on IABP and 49 ± 35 off IABP (*p* = 0.41) in aortocoronary grafts. Graft flow on IABP was more than 5 ml/min greater in 28 (37%) bypass grafts, and more than 5 ml/min lower in 20 (26%) bypass grafts.

**Conclusion:**

In contrast to previous reports for conventional CABG, graft flow after OPCAB was not necessarily increased by IABP, regardless of elevated diastolic arterial pressure. It is suggested that preserved autoregulation of coronary flow contributes to a lower impact on the heart and early functional recovery, and consequently, greater perioperative safety of OPCAB.

## Background

Off-pump coronary artery bypass grafting (OPCAB) is beneficial for patients with systemic comorbidities, such as renal or lung disease, or aortic calcification, or in patients who are elderly. OPCAB was also reported to have less impact than conventional on-pump coronary artery bypass grafting (ONCAB) on cardiac function as determined by cardiac enzyme release [1–3], and allowed earlier recovery of the myocardium after surgery [4, 5]. In addition, subendocardial myocardial damage was frequently detected by magnetic resonance imaging after on-pump beating coronary artery bypass graft (OBCAB) [6]. However, the effects of OPCAB on the myocardium or perioperative coronary circulation have not yet been fully determined.

In the present study, we examined the effects of intra-aortic balloon pumping (IABP) on graft flow after OPCAB, and compared the effects with those after ONCAB to determine the influence of cardiopulmonary bypass on flow regulation and the physiological nature of the coronary circulation after OPCAB.

## Methods

Clinical records and angiograms of 32 patients (25 male, 7 female; mean age: 70 ± 9 years) who underwent OPCAB with IABP between January 2011 and May 2015 at our institution were retrospectively reviewed. This study was approved by our institutional review board. As shown in Table 1, 76 bypass grafts with 102 distal anastomoses were created. Mean targets were 3.2 ± 0.8. Twelve (38%) patients had diabetes mellitus, and 13 (41%) patients had a history of percutaneous coronary intervention.

**Table 1:** Baseline patients' characteristics

No. of patients	32
Age (yrs)	70 ± 9
Male/Female	25/7
Hypertension	22 (69%)
Hyperlipidemia	9 (28%)
Diabetes	12 (38%)
Renal dysfunction (Creatinin > 2.0)	9 (28%)
History of percutaneous coronary intervention	13 (41%)
History of myocardial infarction	13 (41%)
History of stroke	9 (28%)
Ejection fraction of left ventricle (%)	45 ± 15
Ejection fraction of left ventricle < 40%	11 (34%)
Total distal anastomoses	102
Targets per patient	3.2 ± 0.8

Conduit types are listed in Table 2. There were 50 in situ (pedicled) grafts, including 38 in situ internal thoracic artery (ITA), 3 in situ gastroepiploic artery, and 9 composite grafts, and 26 aortocoronary grafts, including 25 saphenous vein grafts and one free ITA. The indication for IABP was unstable angina pectoris following acute or recent myocardial infarction in 11, unstable angina without myocardial infarction in 20. In one patient, acute myocardial infarction was due to coronary dissection during coronary intervention. Patients, who presented cardiogenic shock, necessitated cardiopulmonary bypass were excluded. In preoperative coronary angiography, TIMI grade II antegrade or reduced collateral flow was found in 6 coronary vessels of 5 patients. Operation was emergent or urgent in all but one patient, who had poor left ventricular function resulting from an old myocardial infarction. No patient had atrial fibrillation.

**Table 2:** Conduit types

Aortocoronary	Saphenous vein graft	25 (33%)
	Free ITA	1 (1%)
In-situ (pedicled)graft	In-situ ITA	38 (50%)
	Composite graft	
	in-situ ITA with free ITA	8 (11%)
	in-situ ITA with radial artery	1 (1%)
	In-situ GEA	3 (4%)
Total		76 (100%)

*GEA* gastroepiploic artery, *ITA* internal thoracic artery

Details of the OPCAB procedure and routine flow measurement have been reported previously [7]. After completion of the anastomoses, the position of the heart was returned to normal. Flow measurement using transit-time flowmetry (TTFM; Medi-stim, Oslo, Norway) was started when the haemodynamics became stable, which was usually at a systolic blood pressure greater than 100 mmHg, usually with low dose of noradrenaline and dopamine. First, graft flow under 1:1 IABP support was recorded. Then, IABP was turned off for a period of 30 s to a few minutes. When graft flow became constant, it was recorded. The measurement usually took less than 5 min. The haemodynamic status, such as blood pressure, heart rate, and dose of catecholamine could not be strictly defined, but the haemodynamic status and dose of catecholamine during flow measurement on IABP and off IABP were comparable in each patient. For sequential or composite bypass grafts, graft flow was measured in the proximal graft. For patients without renal dysfunction or other comorbidity, such as severe calcification of the aorta or severe chronic obstructive pulmonary disease, catheter or computed tomography coronary angiography was performed about 2 weeks after surgery.

### Statistical analysis

The continuous variables are expressed as mean ± standard deviation and compared with the unpaired Student *t*-test, using SPSS version 8.0 (SPSS Inc., Chicago, IL, USA). Differences in outcomes were considered statistically significant when at *p* < 0.05.

## Results

**Fig. 1:**
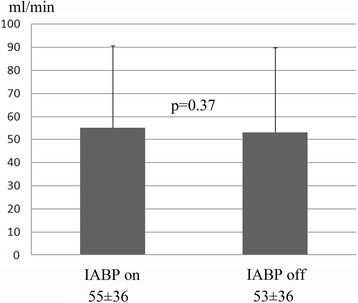
Mean graft flow during on IABP (left) and off IABP (right). There was no significant difference (*p* = 0.37)

**Fig. 2:**
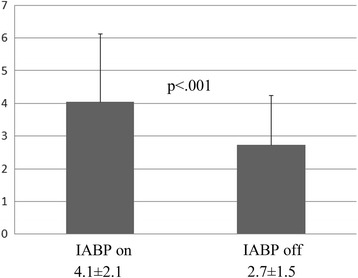
Pulsatility Index during on IABP (left) and off IABP (right). PI on IABP was significantly higher than that during off IABP (*p* < .001)

In postoperative angiography, the graft patency rate was found to be 100% (47/47). Mean graft flow was 55 ± 36 ml/min on IABP and 53 ± 36 ml/min off IABP (Fig. 1). Flow was 3.8% higher on IABP but the difference was not statistically significant (*p* = 0.37). The pulsatility index on IABP was 4.1 ± 2.1, and was significantly higher than off IABP (2.7 ± 1.5; *p* < 0.001) (Fig. 2).

In situ grafts and aortocoronary bypass grafts were analysed separately. There was no significant difference in graft flow between on and off IABP for aortocoronary bypass or in situ grafts. Mean graft flow was 57 ± 36 ml/min on IABP and 55 ± 37 ml/min off IABP (*p* = 0.41) in in situ grafts, and 52 ± 34 ml/min on IABP and 49 ± 35 ml/min off IABP (*p* = 0.41) in aortocoronary grafts. The pulsatility index was 3.8 ± 1.5 on IABP and 2.5 ± 1.0 off IABP in in situ grafts (*p* < 0.001) and 4.6 ± 2.9 on IABP and 3.3 ± 2.1 off IABP in aortocoronary bypass grafts (*p* < 0.001).

**Fig. 3:**
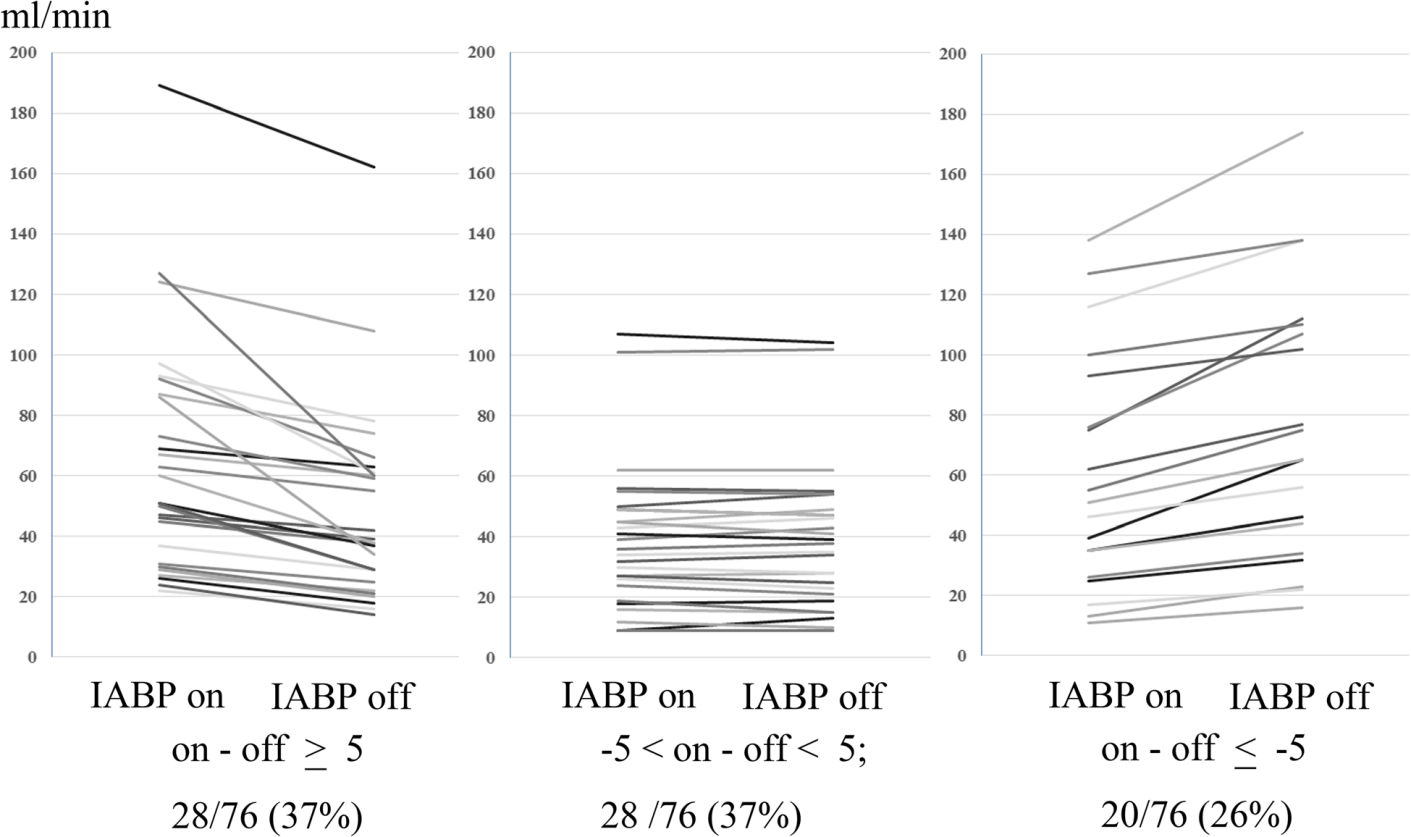
Changes of mean graft flow during on IABP and off IABP. Graft flow on IABP was more than 5 ml/min greater in 28 (37%) bypass grafts (*left*), and more than 5 ml/min lower in 20 (26%) bypass grafts (*right*), and the difference was less than 5 ml/min in 28 (37%) bypass grafts (*middle*)

Compared with off IABP, mean graft flow on IABP was greater in 42 (55%) bypass grafts, and lower in 32 (42%) bypass grafts. The changes in each patient are shown in Fig. 3. Graft flow on IABP was more than 5 ml/min greater in 28 (37%) bypass grafts, and more than 5 ml/min lower in 20 (26%) bypass grafts.

## Discussion

After OPCAB, patients have been reported to have less myocardial injury as determined by biochemical analyses. Koh and colleagues measured serial troponin T release and reported less damage during OPCAB than ONCAB [1]. Dijk and colleagues reported that creatine kinase-MB release after OPCAB was reduced compared with ONCAB [2]. Although it is difficult to distinguish myocardial stunning from irreversible injury even with biochemical markers, electrocardiography, or functional assessment [3], several studies confirmed that off-pump surgery improved left ventricular contractility early after surgery [4, 5]. In the late follow-up period, there was no significant difference between ONCAB versus OPCAB in the incidence or extent of irreversible myocardial injury [4], or in left ventricular function at 6 months [5].

Cardiac magnetic resonance imaging is reported to be reliable for detecting perioperative small myocardial necrosis [8]. Rahimi and colleagues reported that revascularization-related hyper-enhancement was found in 32% of patients and predicted an adverse cardiac event after percutaneous coronary intervention or coronary artery bypass grafting [9]. Pegg and colleagues reported that the incidence of new irreversible myocardial injury was significantly higher in OBCAB than ONCAB under cardiac arrest [6].

It is generally accepted that IABP has a beneficial effect in reducing cardiac work or myocardial afterload. However, it is unclear whether there is an increase in blood flow through stenotic coronary vessels or increased collateral flow. Williams and colleagues reported that, after IABP in patients who had unstable angina with left anterior descending coronary artery stenosis, O_2_ consumption was reduced, but great cardiac vein flow was also reduced [10]. Yoshitani and colleagues reported that use of IABP decreased diastolic intraluminal pressure distal to the stenosis, especially when the stenosis was severe [11]. In contrast, Takeuchi and colleagues reported a significant increase in coronary flow distal to the stenosis [12].

Several studies have reported that blood flow in bypass grafts was markedly increased by IABP. In an animal model, IABP increased diastolic flow by 75% in aortocoronary bypass grafts and by 38% in ITA grafts [13]. In that experimental study, graft flow was measured after on-pump surgery and cardiac arrest. In a clinical study, Onorati and colleagues found highly significant increases of 50–90% in blood flow in all kinds of grafts [14]. In their report, 78% of patients underwent ONCAB. Rubino and colleagues reported an increase in graft flow of 55–95% with IABP in patients after ONCAB [15]. Takami and colleagues examined graft flow in 84 patients, 45% of who had ONCAB, and reported that mean flow was significantly increased by 23% [16]. In general in these previous studies, the mean increase in graft flow was greater as the proportion of ONCAB increased. Consequently, Onorati and colleagues concluded that a combination of IABP and TTFM were useful for detecting graft failure [14], such that no increase in flow with IABP indicated a failed graft.

However, these results may be contradictory to the concept of “autoregulation”, which is characterised by the adaptability of vascular resistance to changes in blood pressure over a wide range, and is essential for maintaining appropriate flow distribution in different regions and layers with variable vascular resistance, such as the endocardial or epicardial myocardium [17]. In addition, in patients with multivessel disease, vascular resistance in the area of the collateral receiving vessel must be significantly lower than that in the area of the collateral delivering vessel. According to a few papers investigating the effects of IABP in normal coronary arteries, there was an increase in coronary flow of only 1.1% [18] or 11% [19].

Balacumaraswami and colleagues investigated arterial pressure and blood flow in bypass grafts and compared ONCAB with OPCAB [20]. After ONCAB, graft flow was significantly higher for all grafts than after OPCAB, and the flow/pressure ratio was greater for all grafts after ONCAB [20]. The study mentioned that reactive hyperaemia resulting from cardiopulmonary bypass and myocardial ischemia was the mechanism for increased graft flow, in spite of lower arterial pressure. Cardiopulmonary bypass causes not only a systemic inflammatory response and multiorgan dysfunction [21], but also a cardiac hyperaemic state, resulting in impairment of autoregulation. In addition, Ono and colleagues reported that impaired autoregulation of cerebral blood flow by cardiopulmonary bypass was detected in 20% of their patients, and associated with perioperative stroke [22].

In the present study, there was no significant difference in graft flow between on IABP and off IABP after OPCAB, suggesting that OPCAB has specific physiological characteristics. We suggest that autoregulation may be preserved or minimally damaged by OPCAB as there is only localized rather than global cardiac ischemia. In addition, the increase in flow with IABP in proportion to the increase in diastolic pressure in previous studies may be attributable to a “hyperaemic state” induced by cardiopulmonary bypass and cardiac arrest. In the current study, graft flow on IABP was markedly lower than flow without IABP in 26% of bypass grafts. One reason for this is that reduced cardiac work and oxygen demand with IABP could decrease coronary flow, irrespective of higher diastolic pressure. Another possibility is that there is decreased resistance through the stenotic portion of the native coronary artery when diastolic blood pressure increases [23]. Thus, IABP may increase blood flow through the native coronary artery with severe stenosis or the collateral circulation, rather than the bypass graft without stenosis. This would be consistent with the absence of an increase  in pressure in the vessel distal to the severe stenosis, as reported by Yoshitani and colleagues [11].

There are implications arising from this study. As mentioned in previous papers, one explanation for subendocardial damage in OBCAB may be inadequate coronary perfusion to distal territories [6]. We suggest, first, that another possible explanation may be preserved autoregulation after OPCAB, which would function to protect from malperfusion even in coronary circulation with variable vascular resistance. Second, TTFM during OPCAB may reflect a more physiological and natural coronary circulation compared with ONCAB. Therefore, TTFM during OPCAB is considered reliable for detecting not only technical failure, but also future graft occlusion associated with poor graft flow resulting from moderate native coronary stenosis or poor flow demand [24]. Third, even in our limited experience, the patency of a graft is not correlated with an increase or decrease in graft flow with IABP, in contrast to the effect of IABP after ONCAB. Consequently, preserved autoregulation would play a definitive role in the perioperative safety of OPCAB. We suggest that autoregulation may be associated with a lower risk of hypoperfusion syndrome as a result of in situ ITA grafting, even after division of the old saphenous vein graft in redo off-pump surgery. Conversely, there may be a concern over increased risk of coronary spasm during OPCAB because of the absence of vasodilatation by induced hyperaemia.

This study has a few limitations. First, the number of patients was limited. If a larger number of bypass grafts were analysed, differences in graft flow between on IABP and off IABP may have reached statistical significance. However, we consider that any difference would never be as great as those reported in previous ONCAB studies. Second, selection bias might have been present, as most of our patients had unstable angina and urgent or emergency surgery was required. In such situations, coronary flow could be impaired by the underlying myocardial stunning. In addition, preoperative angiography showed coronary flow of TIMI grade II in 5 patients, which might be associated with ischemic damage on microvascular circulation. We presume that, however, increased graft flow during IABP cessation, which was seen in 26% of bypass grafts, could not be explained by microvacular damage or myocardial stunning. These were inevitable limitations of a retrospective observational study in the clinical field. Taking these into account, however, we believe that this study indicates that there is regulation of coronary flow after OPCAB, in contrast to the state after ONCAB.

## Conclusions

In conclusion, graft flow after OPCAB is not necessarily increased by IABP, probably because damage to coronary autoregulation is minimal. In contrast to ONCAB, the absence of an increase in mean flow with IABP was not indicative of possible graft occlusion or the necessity for anastomotic revision. Preserved autoregulation is a possible mechanism and would be consistent with the less invasive nature of OPCAB compared with ONCAB. Further investigations are necessary to confirm these findings.

## Abbreviations

IABP: Intraaortic balloon pumping
ITA: Internal thoracic artery
OBCAB: On-pump beating coronary artery bypass grafting
ONCAB: On-pump conventional coronary artery bypass grafting
OPCAB: Off-pump coronary artery bypass grafting
PI: Pulsatility index
TTFM: Transit-time flowmetry
